# Mismatch induced speciation in *Salmonella*: model and data

**DOI:** 10.1098/rstb.2006.1925

**Published:** 2006-10-11

**Authors:** Daniel Falush, Mia Torpdahl, Xavier Didelot, Donald F Conrad, Daniel J Wilson, Mark Achtman

**Affiliations:** 1 Peter Medawar Building for Pathogen Research, Oxford UniversityOxford OX1 3SY, UK; 2 Danish Institute for Food and Veterinary Research, Department of MicrobiologyBülowsvej 27, 1790 Copenhagen V, Denmark; 3 Department of Human Genetics, University of Chicago920 East 58th Street, CLSC 507, Chicago, IL 60637, USA; 4 Department of Molecular Biology, Max-Planck Institut für Infektionsbiologie10117 Berlin, Germany

**Keywords:** rational systematics, homology-dependent recombination, mismatch repair, genomics, recombination

## Abstract

In bacteria, DNA sequence mismatches act as a barrier to recombination between distantly related organisms and can potentially promote the cohesion of species. We have performed computer simulations which show that the homology dependence of recombination can cause *de novo* speciation in a neutrally evolving population once a critical population size has been exceeded. Our model can explain the patterns of divergence and genetic exchange observed in the genus *Salmonella*, without invoking either natural selection or geographical population subdivision. If this model was validated, based on extensive sequence data, it would imply that the named subspecies of *Salmonella enterica* correspond to good biological species, making species boundaries objective. However, multilocus sequence typing data, analysed using several conventional tools, provide a misleading impression of relationships within *S. enterica* subspecies *enterica* and do not provide the resolution to establish whether new species are presently being formed.

## 1. Introduction

Neutral models are highly valued in population genetics, even by those who do not subscribe to them (Kreitman 1996). They provide a null hypothesis to be falsified and, in doing so, frame most evolutionary analysis of DNA sequence data. Bacterial populations are large, making natural selection a potent force, e.g. moulding codon bias (Hartl *et al*. 1994) and other weakly selected features of genome composition. For this reason, it has been hypothesized that the boundaries between different phenotypic, or even genotypic, clusters of bacteria are principally moulded by selection (Palys *et al*. 1997). However, substantial clustering in both genotype and phenotype spaces is expected even under the simplest neutral models (Felsenstein 1985), making it challenging to prove that selection has really acted and to what effect.

In this paper, we will attempt to expand those features of bacterial population structure that can potentially be explained by purely neutral models. In this way, we reduce the risk that natural selection will be inferred incorrectly. We also aid the selectionist by making the concepts clearer. In particular, we will attempt to show that higher-level divisions, involving genetic barriers to genetic exchange, might be best thought of as arising principally due to neutral processes. Within these higher-level clusters, there might nevertheless be substantial organizing by natural selection, for example, into ‘ecotypes’ that are best defined by the niche that they occupy.

The remainder of this paper is organized into four parts. In §2, we present a neutral model of speciation. This model is based on the laboratory observation in several bacterial genera that the rate at which bacteria incorporate homologous DNA into their genome is greatly reduced by mismatches between the bacteria's own DNA sequence and the sequence of the potential import (Shen & Huang 1986; Zawadzki *et al*. 1995; Zahrt & Maloy 1997; Majewski *et al*. 2000). We show that this mechanism has the potential to create biological species (Cohan 1995; Lawrence 2002)—defined by frequent genetic exchange within species but substantial barriers to genetic exchange between them (Dykhuizen & Green 1991; Vazquez *et al*. 1993)—even in the absence of selective factors. We make an attempt to outline the conditions under which, and the stages by which, speciation can occur in a completely neutral model.

Section 3 presents an attempt to fit the predictions of this model to multilocus sequence typing (MLST) data from different recognized subspecies and species within genus *Salmonella*. We present a plausible, albeit unproven, scenario that could explain the observed pattern of genetic distances between species. In §4, we describe an unsuccessful attempt to use the model in order to identify nascent speciation within the species for which we have the greatest data, namely *Salmonella enterica* subspecies *enterica*. Finally, we discuss the broader implications of our difficulties in fitting model and data in terms of what can and cannot be achieved in classifying species boundaries and mechanisms using MLST data.

## 2. Simulation model

We simulated a neutrally evolving population of circular bacterial genomes of size 10 kb. The bacteria recombine by a homology-dependent process, such that frequent attempts are made to import stretches of sequence from other randomly chosen genomes in the population, but imports are rejected with a probability that depends on the number of DNA sequence mismatches with the existing sequence.

Our model is based on the Wright–Fisher model (Fisher 1930; Wright 1931), which assumes a constant population size *N* and non-overlapping generations. Each generation is formed by choosing *N* genomes, with replacement, from the previous generation. For each genome, a Poisson-distributed number of point mutations (with mean 0.1, corresponding to a mutation rate of 10^−5^) are added at random points along each genome at each generation. Further, a Poisson-distributed number of attempts (with mean 5) are made to copy a randomly chosen stretch of sequence, of geometrically distributed length with mean size of 1000 nucleotides, from a homologous sequence taken from a randomly chosen genome from the population. We used two different rules for homology-dependent recombination. In the first rule, log-linear (Roberts & Cohan 1993), the probability *p* of successful import decreases exponentially with the proportion of sequence differences according to the formula log(*p*)=−300*π*, where *π* is the proportion of nucleotides that differ between the import and the original sequence. In the second rule, minimal efficient processing segment (MEPS; Shen & Huang 1986), imports are accepted only if 150 nucleotides on either end of the sequence are identical.

In order to establish whether the population was segregated into distinct genetically isolated units, we calculated ‘*r*-connectivity’, *r*. We define *β* as the average probability of acceptance of exchanges between any pair of genomes. We can then abstract the population as a set of nodes connected by pairwise values of *β*. *r* is the highest value such that for any two genomes in the population, there exists a path connecting them, in which all values of *β* are greater than or equal to *r*. *r* is a measure of the connectivity of the network, which drops dramatically as speciation occurs.

**Figure 1:**
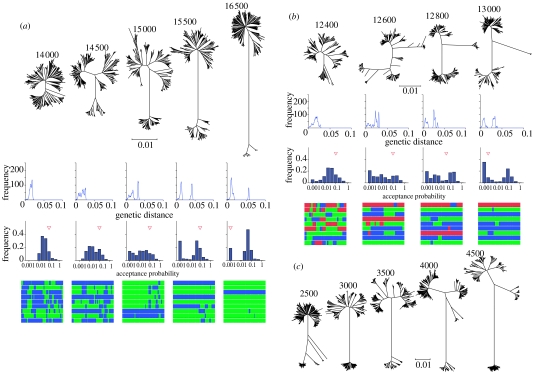
Examples of speciation extracted from simulated bacterial populations. (*a*) A two-way speciation event (movie S2, electronic supplementary material); (*b*) a three-way speciation event (movie S3, electronic supplementary material); (*c*) loss and emergence of species in a large population (movie S4, electronic supplementary material). Each column shows the composition of the population at the time point (generations) indicated above the neighbour-joining tree (calculated using MEGA; Kumar *et al*. 2001). For each time point in (*a*) and (*b*), additional subfigures summarize, from top to bottom, pairwise nucleotide-mismatch distributions, acceptance probability histograms, with an arrowhead indicating the *r*-connectivity, and sources of ancestry for each genome position for nine representative genomes. Sources of ancestry were estimated using the linkage model of Structure, assuming *K*=2 distinct ancestry sources for (*a*) and *K*=3 for (*b*). See the *Salmonella* analysis below for a description of how naive clustering is performed by Structure. The Structure input file contained the genotype of each strain or genome for all nucleotide sites that were polymorphic. Physical distances between adjacent polymorphic nucleotides were input as map distances and each run of structure consisted of a burn-in phase of 2000 iterations, followed by 5000 subsequent iterations. The population size, *N*, is 1000 in (*a*) and (*b*), and 2000 in (*c*). (*a*) and (*c*) were simulated according to a log-linear homology rule, while (*b*) was simulated according to a MEPS rule. For the log-linear rule, the average acceptance probability of imports between a pair of strains is estimated by averaging the import probability of 1000 bp stretches for all genome positions. For the MEPS rule, the probability is estimated by squaring the proportion of the two genomes that are identical for runs of 150 nucleotides or more.

The qualitative behaviour of the model depends on the population size *N*. When *N*=500, the population persists indefinitely as a single biological species (electronic supplementary material, movie S1), with *r* always greater than 0.1. When the population size is increased to *N*=1000, speciation occurs through a complex and highly variable stochastic process (electronic supplementary material, movie S2). Distinct clades emerge repeatedly. Most clades go extinct as a consequence of genetic drift before a genome-wide barrier to genetic exchange can develop. However, occasionally, a clade diverges from the rest of the population to the extent that recombination becomes very rare (e.g. figure 1*a*), reducing the value of *r* for the population to 0.0001 or less.

In our simulations with a population of size *N*=1000, new species disappeared frequently owing to genetic drift and the entire population never contained more than three genetically isolated groups at any one time (figure 1*b*). However, with *N*=2000, sequential events lead to the simultaneous occurrence of multiple species (figure 1*c*; electronic supplementary material, movie S3). The two different types of homology rule that we tested (log-linear and MEPS) gave qualitatively similar results for various parameters, but a population evolving under less strong homology dependence requires either a larger population size or a higher mutation rate to induce speciation (data not shown).

**Figure 2:**
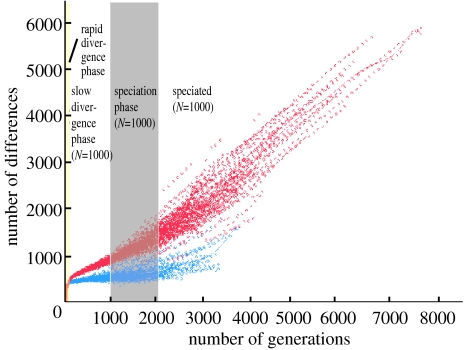
Effect of population size on divergence. Twenty independent simulations with *N*=500 (blue) and 20 independent simulations with *N*=1000 (red) were used in this graph. For each pair of genomes in each simulation and every 100 generations, the time to the most recent common ancestor (TMRCA) and the number of genetic differences were recorded. However, because deep branches of the genealogy correspond to many pairs of individuals, only one pairwise genetic distance, calculated for a randomly chosen pair of individuals, is shown for each possible value of the TMRCA in each generation of each simulation.

Insight into the effect of population size on speciation is provided by the relationship between the number of generations since the common ancestor of a pair of strains and the genetic distance between them (figure 2). For both *N*=500 and 1000, the initial phase of divergence is rapid, with strains picking up multiple sequence differences by recombining with unrelated strains from the same species (this phase occurs approximately between 0 and 100 generations in figure 2). As the divergence between strains increases, the net effect of recombination changes, so that eventually it acts to homogenize rather than to speed differentiation. For *N*=500, the initial divergence gives rise to a stationary phase, in which the divergence between strains is approximately constant. For a few pairs of clades, there is a tendency for the genetic distance to increase slowly over time, which is a hallmark of nascent speciation; however, because the population size is small, one of the two clades generally drifts to extinction before becoming clearly identifiable as a distinct new species.

For *N*=1000, the period of initial rapid divergence is followed by a period in which the nucleotide sequence of the strains diverge from each other at a slow but approximately uniform rate, despite a significant homogenizing effect of recombination. The difference from *N*=500 occurs, because there is greater diversity in the overall population, which leads to lower recombination rates. This phase lasts approximately between 100 and 1000 generations for all pairs of clades. If both the clades persist for long enough in the population, this phase is inevitably followed by the evolution of strong barriers to genetic exchange and speciation. However, the time at which speciation occurs is highly variable, happening after between approximately 1000–2000 generations shown in figure 2. Once speciation has occurred, divergence tends to a constant rate, equal to twice the mutation rate, until one or other of the clades disappears from the population owing to genetic drift.

The population sizes that we have simulated are smaller than reality, even considering effective population sizes rather than census ones, for essentially any bacterial population. Using this type of explicit forward simulation of each nucleotide of each individual in the population and pairwise statistics such as *r*, it is simply not possible to simulate realistic population sizes. The results that we have obtained could potentially be compared directly to real populations, if other parameters are accurate and if both are scaled in terms of coalescent parameters *θ* (the mutation rate scaled in terms of the effective population size) and *ρ* (the recombination rate scaled according to the effective population size). For example, if the mutation and recombination rates are divided by 10 000, and the population size is multiplied by 10 000, then we would expect to get very similar results, with parameter values that would then be much closer to those we might expect for bacterial genera like *Salmonella*. However, it should be noted that it has not been demonstrated formally that this coalescent scaling works for a homology-dependent recombination process and we make only a minimal attempt to fit the model parameters to the *Salmonella* data in §3. One alternative approach is to make approximations based on coalescent theory (Cohan 1995), but this has the disadvantage of not revealing properties of the population at an individual level. Simulation of samples from a large population using the ancestral recombination graph (Wiuf & Hein 2000), which considers the genealogy of each site in a sample, backwards in time, is likely to require a great deal of computation if recombination rates are high and would need to be modified to allow for homology dependence. We do not attempt this here.

These simulations show that frequent homology-dependent recombination leads to specific patterns of variation among organisms. First, related genotypes are organized into a relatively small number of discrete species. Within each species, recombination destroys clonal frames, leading to highly mosaic patterns of ancestry (figure 1*a*,*b*). Second, diversity is essential for speciation, as is a population size that is large enough to generate that diversity. Third, species are monophyletic at the nucleotide level and share few polymorphisms. As a result, species diverge from each other by point mutations, which can obey a molecular clock. Finally, there is a grey zone, where genetic barriers to genetic exchange are developing but are not fully formed. Within this zone, the term ‘fuzzy species’ (Hey 2001; Hanage *et al*. 2005) might be apposite.

## 3. Application to data from *Salmonella* subspecies

The genus *Salmonella* has been divided into seven groups on the basis of DNA/DNA hybridization experiments in combination with biotyping (Le Minor *et al*. 1982*b*). One group, *Salmonella bongori*, is thought to represent a distinct species, while the other six groups (*enterica*, *arizonae*, *diarizonae*, *houtenae*, *indica* and *salamae*) have been designated as subspecies of *S. enterica* (Le Minor *et al*. 1982*a*; Reeves *et al*. 1989; Tindall *et al*. 2005). For simplicity, we refer to each of these designated subspecies by an unadorned subspecies name, e.g. *enterica*.

**Figure 3:**
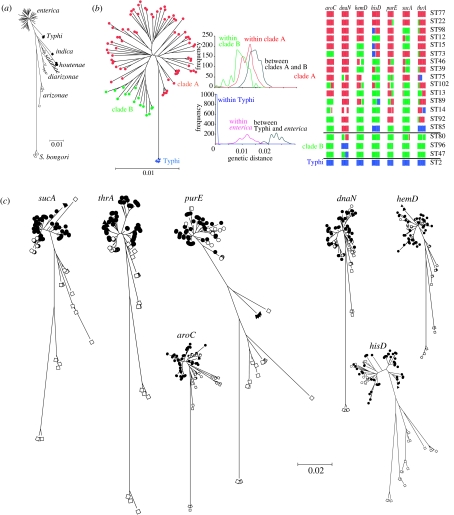
Sequence data within *Salmonella*. (*a*) Neighbour-joining tree of genotypes within *Salmonella*. (*b*) Neighbour-joining tree of genotypes, mismatch distributions between genotypes and Structure analysis of sources of ancestry within *enterica*. Both the neighbour-joining tree and the Structure analysis identified three groups: clade A (red); clade B (green); and Typhi (blue). However, clades A and B are only weakly differentiated, as indicated by intermediate bootstrap support (60%) and between-clade distances, extensive allele sharing and a continuum of ancestry. (*c*) Neighbour-joining trees shown for each MLST fragment. Sequences from strains from clade A are shown as filled circles, from clade B as open circles, from Typhi as filled triangles and from other subspecies or *bongori* as open squares.

The details of the *Salmonella* strain collection are described elsewhere (Torpdahl *et al*. 2005). Seven fragments from housekeeping genes, representing a total of 3336 nucleotides, were sequenced as described (Kidgell *et al*. 2002) and are available at http://web.mpiib-berlin.mpg.de/mlst. We sequenced 207 *enterica*, of diverse serotype, along with 20 strains from other subspecies and *S. bongori* yielding a total of 108 distinct sequence types (STs). Of these, 95 are from *enterica*, with one to four STs from each of the other subspecies and *S. bongori*. The most diverse of the subspecies is *enterica* (table 1) and contains a majority of the 2500 recognized serotypes (Popoff 2001). Neighbour-joining trees based on concatenated sequences confirmed the monophyly of each of the seven groups (figure 3*a*; Selander *et al*. 1996) with 100% bootstrap support (note that Typhi is part of *enterica*; see §4).

*Salmonella* has traditionally been thought of as largely clonal, based on analyses of its population structure by multilocus enzyme electrophoresis (MLEE; Beltran *et al*. 1988; Maynard Smith *et al*. 1993) and nucleotide sequence comparisons (Selander *et al*. 1996). However, this clonal paradigm has recently been questioned (Kotetishvili *et al*. 2002; Brown *et al*. 2003) because phylogenetic trees for several genes within *enterica* are incongruent, indicating that recombination has occurred on multiple occasions. Our data also provide evidence for substantial recombination within *enterica*, as evidenced by a highly mosaic pattern of ancestry (figure 3*b*), with substantial allele sharing between clades.

In contrast to the pattern observed within *enterica*, recombination between *enterica* and the other subspecies is very rare. We used the linkage model of Structure (Pritchard *et al*. 2000; Falush *et al*. 2003) to try and identify even very short imports from the other subspecies. Structure assumes that each strain in the sample draws its ancestry from one of the *K* populations, where *K* is an integer specified by the user that can be varied from run to run. We used the linkage model, meaning that nucleotides are assumed to be inherited in chunks from each ancestral population. Structure assumes that within each of the *K* ancestral populations there has been frequent recombination, so that there is no linkage disequilibrium between nucleotides found in chunks inherited from the same population. Structure can be used to perform naive clustering, such that the composition of the ancestral populations is estimated at the same time as the ancestry of each isolate (see below). Alternatively, it is possible to assign individuals to populations at the outset using the Usepopinfo option and to estimate the ancestry of the remaining isolates conditional on the fixed assignments. We used this option to try and identify imports from the other subspecies into *enterica* (we considered Typhi isolates separately for erroneous reasons; see §4). Each *enterica* strain was also initialized to its own population (using the Startatpopinfo option), but was subsequently allowed to have mixed ancestry during the run. Stretches of sequence that were assigned to *enterica* plus Typhi with less than 50% probability represent putative imports and were checked manually. All Structure runs for the *Salmonella* data were performed with a burn-in of 10 000 iterations and 100 000 subsequent iterations.

We were able to detect only two putative sequence imports from other subspecies in 91 *enterica* genotypes in our sample. First, six *enterica* STs (20, 65, 79, 80, 91 and 94) have similar *aroC* sequences to *arizonae*, presumably reflecting a recent import. Second, three STs (65, 81 and 102) contain an A nucleotide at *hisD*_330_ and *hisD*_333_, whereas all other *enterica* STs possess T and G, respectively, at the two sites. We interpret this observation as most probably representing ancient polymorphism, because the A nucleotide is uniform throughout all the other subspecies and 50 bp flanking these nucleotides in the three STs were typical of *enterica*. Owing to the low number of strains in our sample from other subspecies, we could not use the same tools, but nevertheless inspection of the neighbour-joining trees of the seven gene fragments implies that export from *enterica* to the other subspecies has apparently occurred at a somewhat higher frequency: two genotypes of *arizonae* (STs 55 and 56) and one genotype of *houtenae* (ST 57*)* have *enterica*-like alleles at the *purE* and *sucA* loci, respectively.

Based on the similarities between the patterns of the model (figure 1) and within the multilocus sequence data (figure 3), each of the *Salmonella* subspecies seems to correspond to a genetically isolated biological species. We can construct a sequence of speciation events, consistent with the rules observed during our simulations, that explains the patterns of diversity among the different taxa within the genus. In this sequence, the threshold of diversity within a species that is required to precipitate speciation is approximately 2%. First, in a binary fission event analogous to that observed in figure 1*a*, *S. bongori* split from the common ancestor of each of the subspecies. The genetic distances between *S. bongori* and each of the subspecies are very similar (table 1), consistent with clock-like divergence subsequent to speciation. Subsequently, *arizonae* split from the remaining subspecies in a similar event.

The genetic distances between the remaining five subspecies (table 1) are not consistent with a sequential budding model of speciation. The distances fall within a narrow range (2.9–4.4%), which does not allow a sufficient time window for the first species to have become reproductively isolated before the last species starts to bud. Moreover, *salamae* is the closest neighbour of each of the other subspecies, consistent with the pattern observed in a simulation during a three-way speciation event (figure 1*b*). By contrast, sequential budding should lead to the most closely related pair of subspecies having similar levels of divergence with all the others. Thus, the five subspecies appear to have diverged from each other in a multiple-speciation event. However, this analysis is subject to the substantial uncertainty in actual genome-wide rates of divergence based on MLST data (see §5 for further discussion).

The overall pattern (figure 3*a*) is similar to a snapshot of diversity at a single time point taken from our simulations with a large population size (e.g. figure 1*c*). The data obey the predictions of extensive genetic exchange within species and limited exchange between species. Further, according to our reconstruction, all the speciation events involve the ancestor of *enterica*, consistent with the prediction that only diverse species can speciate. Thus, a simple model of neutral divergence with homology-dependent recombination can explain the principal features of variation observed across the entire genus.

In our simulations, we chose parameters such that speciation would occur at approximately 2% sequence divergence, to approximately match the *Salmonell*a data. In fact, laboratory measurements show even stronger homology dependence. For wild-type strains, the barrier between Typhi and another strain of *enterica* is too strong to allow meaningful recombination (Zahrt & Maloy 1997). When the *mutS* gene is knocked out, recombination is increased 100-fold, which is comparable to that assumed in our simulations for strains with that degree of divergence (table 1). Thus, most of the recombination in *Salmonella* may occur when the mismatch repair system is impaired by genetic or other (Matic *et al*. 2000) factors.

## 4. Is speciation occurring within *enterica*? A cautionary tale

Since *enterica* is the most diverse of the subspecies, we might ask whether it is itself speciating, and if so which lineages constitute the incipient species. Initial analysis of data using both a neighbour-joining tree of concatenated sequences and the program Structure, using naive clustering, suggested three different populations, putatively corresponding to partially reproductively isolated gene pools. The first of these consists of a single lineage, containing the human pathogen Typhi, whose strains all share a recent common ancestor (Kidgell *et al*. 2002). Typhi is clearly separated from the other strains on the neighbour-joining tree, with a pairwise genetic distance of 2%. According to the Structure analysis, Typhi is largely reproductively isolated from the other two. All the nucleotides in Typhi derive from a single gene pool (blue in figure 3*b*), which contributes only 7% on average to the ancestry of the remaining *enterica*. Further, and also consistent with this analysis, Typhi shares no identical alleles at any of the seven MLST loci with the remaining *enterica* strains, and it is the only lineage with this property, implying an absence of recent genetic exchange.

The frequency of ancestry from the other two putative gene pools is correlated with whether the strain is in ‘clade A’ or ‘clade B’ in figure 3*b*. These gene pools are less distinct from each other than they are to Typhi according to the observed genetic distances and, indeed, the Structure analysis implies substantial genetic exchange between them. These lineages are apparently at the earliest stages of speciation.

While this analysis is interesting and suggestive, it is completely incorrect, at least with respect to Typhi. The large genetic distance between Typhi and the other subspecies is due entirely to one of the seven gene fragments, *purE*, which is clearly an import into Typhi from another species, with the sequence most closely related to that found in *indica* (figure 3*c*). When this gene fragment is removed, the genetic distances between Typhi and other *enterica* strains are unexceptional. Moreover, genomic analysis of genetic relationships of Typhi with other strains reveals substantial similarities with Paratyphi A according to both gene content (McClelland *et al*. 2004) and sequence data (Didelot *et al*. in press). Typhi no doubt represents a distinct ecotype with some peculiar and interesting properties, but it is not speciating in this biological sense and, in line with the traditional view based on MLEE, is a member of *enterica*.

These analyses are misleading owing to several peculiarities of the dataset. The neighbour-joining tree is misleading because it gives a lot of weight to a single ‘outlier’ gene fragment, which provides little information on the overall genomic composition of Typhi. A fragment-by-fragment bootstrapping procedure would have revealed that the long branch separating Typhi from the rest of *enterica* was not well supported. The pattern of allele sharing provides only information on recent (on the time-scale of the mutation rate) relationships and/or genetic exchange. The absence of shared alleles implies that Typhi has no immediate neighbours within the dataset and has not recombined recently at any of the seven gene fragments, but since this represents a small sample in comparison to both the genome and the time depth of evolution within *enterica*, this observation is of limited informativeness about how Typhi might differ from other lineages. Structure assumes that each of the isolates is unrelated in the sense that they have acquired their genomes independently from a number of distinct ancestral sources. In fact, the four Typhi STs in the sample are closely related to each other by recent common descent. This clonal relatedness, plus the outlier sequence, which includes many fixed differences between Typhi and the other strains, causes Structure to infer that Typhi represents a distinct, recombining population, when in fact it is just a clone.

Given that each of these analysis methods has led us to misleading results about Typhi, it follows that we need to be very careful in reaching conclusions about the status of the subdivision between clades A and B. There are a number of possibilities. Clade B could represent an expanding or disproportionately sampled clone, which accounts for a large proportion of the STs in the database but is not particularly old, and therefore the clades have not accumulated enough specific mutations in order to have acquired a genetic barrier to genetic exchange. Clade B could represent a set of strains that share a common outlier gene sequence at one of the seven gene fragments but are otherwise not particularly related. Finally, clade B could represent an old and genuinely distinct lineage of *enteric*a that is in the initial stages of speciation.

Inspection of the neighbour-joining trees (figure 3*c*) shows that no single gene is responsible for the clustering together of the strains in clade B. Instead, the sequences typically cluster into one or two groups of closely related alleles, which are often also very closely related to sequences in clade A. None of the sequences is an outlier. Thus, clade B is not obviously a recently emerged clone, nor is it an obvious artefact. It may therefore represent the deepest lineage within extant *S. enterica* and also the lineage that is closest to speciating, although a great deal more data would be necessary to confirm this hypothesis.

## 5. Discussion

This paper shows that species boundaries could, and arguably should, be defined based on reproductive isolation in some bacterial genera. Section 2 describes a simple simulation model of short bacterial genomes, which evolve neutrally with homology-dependent recombination. Given appropriate values for the rate of mutation, recombination, strength of homology dependence and effective population size, the model leads to the creation and maintenance of distinct, reproductively isolated species. Within this model, there is a substantial fuzzy zone where species boundaries are developing but not fully established, but otherwise species boundaries can be objectively defined.

Sections 3 and 4 describe an attempt to show that the model could explain the pattern of divergence and exchange in the genus *Salmonella*, as assayed by MLST. In many aspects, this attempt is successful. Subdivisions are found that are entirely consistent with the original species and subspecies boundaries as defined by MLEE. The pattern of divergence between the subspecies is consistent with the most numerous and hence diverse subspecies, namely the ancestor of *enterica*, repeatedly speciating, as predicted by the model. Moreover, in the subspecies for which we have most data (*enterica*), there is evidence for substantial within-species recombination. If this interpretation of the MLST data was correct, it would imply that the subspecies are biological species and hence deserve full species status.

To decide whether this interpretation is correct will need sufficient data that a systematic approach both reliably yields a consistent outcome in terms of where the species boundaries are drawn and demonstrates that the historical pattern of divergence and reproductive isolation fits the qualitative predictions of the model. These criteria are not fulfilled by the present study. Using a naive approach based on MLST and not relying on previous species definitions, several different analysis methods would imply that Typhi is a distinct species, a result that is shown to be entirely spurious and principally owing to a single import of DNA from another subspecies that leads to an atypically high level of nucleotide differences with other *enterica* at one of the seven MLST fragments. It is also not clear, based on MLST, whether an additional subdivision within *enterica* into clades A and B represents a genome-wide pattern and whether any degree of reproductive isolation has evolved between members of the two clades. It is therefore not clear whether these clades are fuzzy species on their way to speciating, reflect the presence of a genuine but unremarkable clonal lineage within *enterica*, or are simply the result of fuzziness in the data owing to the limited amount of DNA surveyed. Evidence is also lacking as to whether the rate of recombination is sufficient to maintain the cohesion of the other less diverse subspecies.

In summary, the data are consistent with there being a diversity threshold for speciation in *Salmonella*, with the subspecies representing biological species, but the evidence is presently inconclusive. While MLST data have confirmed and extended observations made by MLEE, a genomic view of relatedness between strains will be required to fully apply biological or other model-based species concepts to bacteria.

## Figures and Tables

**Table 1:** Genetic distances within and between STs for all *Salmonella* taxa. (Each cell shows the average nucleotide distances (above) and the average recombination acceptance probability according to the simulated log-linear model (below). The number of STs for each taxon is indicated in the first column. Note that *enterica* here excludes Typhi.)

	*enterica*	Typhi	*salamae*	*indica*	*diarizonae*	*houtenae*	*arizonae*	*S. bongori*
*enterica* (91)	0.012	0.022	0.032	0.036	0.044	0.044	0.061	0.104
0.1	0.03	0.008	<0.001	<0.001	0.001	0.002	<0.001
Typhi (4)		0.0004	0.029	0.034	0.041	0.040	0.067	0.106
	0.8	0.004	0.001	<0.001	<0.001	<0.001	<0.001
*salamae* (3)			0.006	0.028	0.028	0.029	0.060	0.102
		0.3	0.006	<0.001	0.002	<0.001	<0.001
*indica* (1)					0.039	0.039	0.064	0.110
				<0.001	<0.001	<0.001	<0.001
*diarizonae* (4)					0.009	0.042	0.067	0.111
				0.6	<0.001	<0.001	<0.001
*houtenae* (3)						0.002	0.067	0.105
					0.6	<0.001	<0.001
*arizonae* (3)							0.014	0.106
						0.4	<0.001
*S. bongori* (3)								0.004
							0.5

## Supplementary Material

Mismatch distributions and acceptance probabilities during a long simulation run: population size 500 Based on simulations incorporating a log-linear homology rule

Mismatch distributions and acceptance probabilities during a long simulation run: population size 1000 Based on simulations incorporating a log-linear homology rule

Mismatch distributions and acceptance probabilities during a long simulation run: population size 1000 Based on the MEPS model

Mismatch distributions and acceptance probabilities during a long simulation run: population size 2000 Based on simulations incorporating a log-linear homology rule
